# Listening to health workers: lessons from Eastern Uganda for strengthening the programme for the prevention of mother-to-child transmission of HIV

**DOI:** 10.1186/1472-6963-12-3

**Published:** 2012-01-05

**Authors:** Joseph Rujumba, James K Tumwine, Thorkild Tylleskär, Stella Neema, Harald K Heggenhougen

**Affiliations:** 1 Department of Paediatrics and Child Health, College of Health Sciences, Makerere University, P.O.Box 7072, Kampala, Uganda; 2 Centre for International Health, University of Bergen, Bergen, Norway; 3 Department of Sociology and Anthropology, College of Humanities and Social Sciences, Makerere University, Kampala, Uganda

## Abstract

**Background:**

The implementation and utilization of programmes for the prevention of mother-to-child transmission (PMTCT) of HIV in most low income countries has been described as sub-optimal. As planners and service providers, the views of health workers are important in generating priorities to improve the effectiveness of the PMTCT programme in Uganda. We explored the lessons learnt by health workers involved in the provision of PMTCT services in eastern Uganda to better understand what more needs to be done to strengthen the PMTCT programme.

**Methods:**

A qualitative study was conducted at Mbale Regional Referral Hospital, The AIDS Support Organisation (TASO) Mbale and at eight neighbouring health centres in eastern Uganda, between January and May 2010. Data were collected through 24 individual interviews with the health workers involved in the PMTCT programme and four key informants (2 district officials and 2 officials from TASO). Data were analyzed using the content thematic approach. Study themes and sub-themes were identified following multiple reading of interview transcripts. Relevant quotations have been used in the presentation of study findings.

**Results:**

The key lessons for programme improvement were: ensuring constant availability of critical PMTCT supplies, such as HIV testing kits, antiretroviral drugs (ARVs) for mothers and their babies, regular in-service training of health workers to keep them abreast with the rapidly changing knowledge and guidelines for PMTCT, ensuring that lower level health centres provide maternity services and ARVs for women in the PMTCT programme and provision of adequate facilities for effective follow-up and support for mothers.

**Conclusions:**

The voices of health workers in this study revealed that it is imperative for government, civil society organizations and donors that the PMTCT programme addresses the challenges of shortage of critical PMTCT supplies, continuous health worker training and follow-up and support for mothers as urgent needs to strengthen the PMTCT programme.

## Background

Mother-to-child transmission (MTCT) is the leading source of HIV infection in children under 15 years [1]. MTCT refers to HIV infection transmitted from an HIV-infected mother to her child during pregnancy, labour, delivery or breastfeeding [1]. In Uganda, MTCT accounts for 21% of the total HIV transmission[2,3]. The programme for prevention of mother-to child transmission of HIV (PMTCT) in Uganda commenced in 2000 and has since been expanded over the years [4]. By June 2009, 77% of all health facilities in Uganda, from hospitals down to health centre III, were offering PMTCT services, compared to 53% of the health facilities in the same categories in 2008. Despite the scale-up of the PMTCT programme in Uganda, the utilisation of PMTCT services by women living with HIV remains low [4], estimated at 52% in 2009.

One of the weaknesses cited by scholars to explain the undesired performance of PMTCT programmes in low-income countries, such as Uganda, relates to health system failures like poor health worker performance. Health workers have largely been depicted as a weakness in health care delivery with some studies indicating that they have negative attitudes towards their clients [5-9]. An evaluation of PMTCT services in Uganda done in 2003, when HIV testing was being offered as a voluntarily service, documented challenges related to staff, space and reluctance of women to test for HIV [10]. The views of, and lessons learnt by, health workers are important in generating ideas on how to improve the effectiveness of the PMTCT programme in Uganda, more so, within the current context where HIV counselling and testing services as part of the PMTCT programme are integrated into maternal and child health care services [11,12]. We explored the lessons learnt by heath care workers involved in the delivery of PMTCT services in Mbale District eastern Uganda on what more needs to be done to strengthen the programme.

## Methods

### Study design

Between January and May 2010, we conducted a cross-sectional qualitative exploratory operational research study in Mbale District eastern Uganda. Operational research denotes search for knowledge on interventions, strategies, or tools that can enhance the quality, effectiveness, or coverage of programmes about which the research is being done [13,14] in our case the PMTCT programme. A qualitative study design was adopted for being appropriate in understanding social processes and concepts from the perspectives of study participants (health workers), informed by their lived experiences [15] in implementing the PMTCT programme.

### Study area

The population of Mbale District is estimated at 416 600 people [16]with the majority (90%) residing in rural areas [17]. Mbale District has one regional referral hospital which doubles as the district hospital and 40 health centres all providing HIV services at different levels [16]. The study was conducted among health workers involved in the delivery of PMTCT services at Mbale Regional Referral Hospital, eight selected health centres in Mbale District and at The AIDS Support Organisatition Mbale (TASO). Five out of the ten health care facilities provided maternity services and the remaining five health facilities referred mothers for maternity care to the neighbouring health facilities, mainly Mbale Regional Referral Hospital. Selection of health facility was done purposively based on: provision of PMTCT services and variations in the level of health facilities as well as length of implementation of PMTCT services (Table 1).

**Table 1:** Characteristics of study health facilities

Name and level of study Health facility^d^	Year when PMTCT services started^a^	Provides maternity Services
Mbale Hospital	2002	Yes
Bufumbo HC IV	2005	Yes
Bukedea HC IV**^b^**	2005	yes
Iki Iki HC III	2008	yes
Nakaloke HC III	2008	yes
Maluku HC III	2008	no
Namatala HC III	2008	no
Namakwekwe HC III	2008	no
Busamaga HC III	2008	no
TASO Mbale**^c^**	2008	no
**Total**		**10 health facilities**

^a ^PMTCT services that were provided include HIV testing, nevirapine for mothers and babies. All health facilities were referring infants for early HIV diagnosis to the Joint Clinic Research Centre (JCRC) in Mbale town.

^b ^Hosts the largest monthly TASO outreach clinic where PMTCT and ART services are provided

^c^TASO does not have her own maternity services but refers pregnant women in the PMTCT programme to nearby health facilities for delivery mainly Mbale Hospital.

**^d ^**The average monthly new ANC attendance was 700 for Mbale hospital, 200 for TASO, and ranged between 180-200 for HC IVs and 55-70 for health centre IIIs.

Three of the ten health facilities (TASO, Mbale Hospital and Bukedea) had unique characteristics. Mbale Hospital was included in the study because of being the main hospital that offers PMTCT services in the district and a major referral hospital for maternity services for the TASO PMTCT programme and neighbouring health centres. Bukedea Health Centre IV is one of the largest TASO outreach sites where people living with HIV are offered counselling and ARVs on an outreach basis once a month. During the outreach clinic, women in the PMTCT programme are provided with follow-up counselling on ARVs for prophylaxis (for mothers and babies), infant feeding, place of delivery, disclosure of HIV status and are linked to TASO community counsellors for further support.

TASO Mbale was studied because it is one of the major HIV care centres in the district and is one of the 11 TASO Uganda service centres widely spread in the country [18]. TASO Mbale serves clients from the districts of Mbale, Sironko, Manafwa, Bududa, Pallisa, Budaka, Bukedea, Budadiri, Kumi, Butaleja and Kapchorwa in eastern Uganda. The TASO centre provides clinic based, outreach and community based HIV and AIDS services. By the end of 2009, TASO Mbale had registered a cumulative number of 22 500 clients out of which 3 893 were on ART [19]. The PMTCT programme at health facilities in Mbale District was mainly supported by the Government of Uganda and Protecting Families Against HIV/AIDS (PREFA), a non governmental organization that provides support for PMTCT programmes in selected Ugandan districts since 2008. PREFA was funded by the Centers for Disease Control (CDC, Atlanta) and the Children's Investment Fund Foundation (CIFF). In Mbale District, PREFA has been supporting the PMTCT programme since 2008 and was providing supplementary HIV test kits, nevirapine, cotrimoxazole, health worker training, meeting costs for CD4 tests and infant diagnosis, as well as employing two midwives and two counsellors. Support from PREFA ended in March 2010. The PMTCT programme at TASO was receiving funding from the Presidential Emergence Plan for AIDS Relief (PEPFAR).

### Study participants

The study participants were health workers involved in the delivery of PMTCT services at ten selected health care sites in Mbale and Bukedea Districts. The health workers included medical doctors, clinical officers, nurses/midwives and counsellors. Study participants were selected purposively on the basis of: having worked at the study health facility for at least 6 months, was working in the PMTCT clinic at the time of the study, was present on the day of interview and provided consent to participate in the study. Overall, 24 health workers participated in the study. In addition, two district officials from the Department of Health Mbale and two administrators from TASO Mbale participated in the study as key informants. Interviews with health workers were conducted first and those with key informants later. This allowed us to follow up on the suggestions made by health workers on how to strengthen the PMTCT programme with administrators at the district and at TASO. At the end of the interviews, we gave feedback on the major gaps identified at study sites to district officials for their action.

### Data collection methods

#### Individual interviews with health workers

A semi-structured interview guide [20] was used to conduct interviews with the health workers. The semi-structured interview guide consisted of structured questions on: category/position of respondent, training on PMTCT, and length of time the health worker had been involved in activities and services related to PMTCT. These were followed by open ended questions on health workers' perspectives with regard to: routine counselling and testing, and what could be done to strengthen the PMTCT programme. In this paper we present findings from the last part of the interview. The first author (JR) together with three research assistants, all university graduates of social sciences and with experience in qualitative research, conducted the interviews. To enhance quality of the data collected, the research assistants were trained for one day on data collection tools, interviewing techniques and note taking. They participated in the pre-test of study tools for another one day and worked with the first author as note takers on rotational basis. The researchers conducted interviews in pairs, one as an interviewer and the other as a note taker. Pairing of researchers helped to capture detailed notes. As all interviewees were fluent in English, the interviews were all conducted in English and each lasted for about 30-45 minutes. During the interview, the interviewer and the note taker took notes and met at the end of each interview to compile detailed scripts. On average, the number of health workers per site ranged from one to six, the highest being from Mbale Hospital and TASO. These interviews were not audio recorded as health workers were not comfortable with this.

### Key informant interviews

Four officials (2 from Mbale District health department and 2 from TASO Mbale) involved in the planning, implementation and monitoring of the PMTCT programme participated in the study as key informants. An open-ended interview guide was used to collect data on the general state of PMTCT services in the district pertaining to: HIV counselling and testing, maternity, ARVs for prophylaxis and what needs to be done to improve the programme. The first author (JR) conducted the key informant interviews together with one research assistant as a note taker. These interviews were not audio recorded and the first author together with a research assistant compiled detailed notes at the end of each interview.

### Observation during PMTCT clinic days

To gain more understanding of the PMTCT programme and the context within which health workers provided the PMTCT services, the first author (JR) and research assistants attended and made observations during typical PMTCT clinic days at TASO Mbale, TASO Bukedea outreach and Mbale Regional Referral Hospital. The observations were not structured. The researchers attended all activities, including health education talks and group counselling sessions. The research assistants were briefed to note anything that should be replicated, or improved, to strengthen the PMTCT program.

### Data analysis

Preliminary data analysis occurred concurrently with data collection. At the end of each day of data collection, a research team meeting was held to share emerging issues and identify areas for further data collection. In addition, investigators (JR, JT and TT) met in the field twice during data collection to share insights on emerging issues from the study which also informed plans for further data collection and analysis. At the end of the data collection JR held de-briefing meetings with each of the co-authors to share preliminary insights. Further data analysis was conducted manually by JR in close collaboration with HKH. This process of continuous data analysis further assisted in identification of critical themes for the study.

Data were analyzed using the content thematic approach, which was guided by the Graneheim and Lundman 2004 framework [21] to capture latent and manifest content in the interview scripts. Study themes and sub-themes that emerged during data collection, were refined following multiple readings of interview transcripts. In addition to content thematic analysis, we conducted sub-group analysis which involved comparing findings from health workers and key informants as well as those from health workers in public health facilities and TASO. Selected voices of health workers and key informants have been used in presentation of study findings. The identities of individual study participants were masked, for instance we use "health worker, Mbale hospital" to mean all study participants from Mbale hospital, or "health worker, health centre A or B", and so on to denote study participants from health centres.

### Ethical considerations

Ethical approval was obtained from the Centre for International Health, University of Bergen, Norway, Uganda National Council for Science and Technology, Makerere University, College of Health Sciences, Research and Ethics Committee, The Mbale Regional Referral Hospital Institutional Review Committee and the TASO Uganda Institutional Review Board. Permission was also obtained from management at each of the study institutions. Written consent to participate in the study was obtained from all study participants.

## Results

### Social demographic characteristics of study participants

Most of the health workers interviewed were female (17/24) and nurses/midwives (15/24). Only four of the health workers were counsellors only (Table 2) while the rest of the health workers, mainly nurses and midwives, provided counselling as an additional responsibility.

**Table 2:** Characteristics of health workers providing PMTCT services in Mbale District Eastern Uganda

Characteristic	Frequency n = 24 n (%)
**Sex**	
Male	07 (29)
Female	17 (71)
**Health worker category**	
Doctor	02 (08)
Clinical officer	03 (13)
Nurse/midwife	15 (62)
Counsellor	04 (17)
**Length of involvement in PMTCT**	
Less than 2 years	04 (17)
2-5 years	15 (62)
More than 5 years	05 (21)
**Ever attended training on PMTCT**	
Yes	21 (87)
No	03 (13)
**Would like more training on PMTCT **(all yes)	24 (100)
**Respondents by level/type of health facility**	
Mbale Hospital	6(25)
TASO	5(21)
HC IVs	3(12)
HC IIIs	10(42)

### Emerging themes for strengthening the PMTCT programme

The lessons for strengthening the PMTCT programme were grouped under: 1) ensure constant supplies for PMTCT, 2) ensure availability of skilled and up-to-date health workers, 3) provide support for mothers beyond HIV testing, 4) ensure adequate integration and universal rollout of PMTCT services, 5) deal with the challenge of continuing HIV stigma and 6) address heavy work load of health workers (Table 3).

**Table 3:** Emerging themes and sub-themes in the study

Themes	Sub-themes
Ensure constant availability of supplies for PMTCT	Provide requirements for health workers to offer PMTCT services - HIV test kits, ARVs for mothers and babies
Ensure availability of skilled and up-to-date health workers (HWs)	Re-fresher training for all health workers when PMTCT policies and guidelines change
Provide support for mothers beyond HIV testing	Psycho-social support for women through support groups and follow-up visits by health workers.
Ensure adequate integration and universal rollout of PMTCT services	Avail maternity services at lower health facilities, provide ARVs for mothers in ANC
Deal with the challenge of continuing HIV stigma	Provide ARVs and other drugs within the maternal and child health clinics
Address heavy work load of health workers	Adequate number and categories of HWs, supportive PMTCT aids - IEC materials

### Ensure constant availability of supplies for PMTCT

Health workers stressed the need to ensure constant availability of "critical" supplies for PMTCT. These included HIV testing kits, antiretroviral drugs for mothers and their babies, maama kits (supplies used during birth like gloves, cotton wool, and polythene sheets). Study participants reported that the very supplies that define the PMTCT programme were often out of stock. Health workers spoke passionately about how they and their clients (the mothers) had been let down by the shortages, especially kits for HIV testing and ARVs for prophylaxis for mothers and babies.

*I can't say we are doing well. We sometimes lack test kits, Nevirapine for mothers and that for babies. These are the things that make PMTCT. We are even worried that things may get worse because PREFA which has been giving us most of these supplies is closing this month (March 2010). For me, government needs to do everything possible to avail us with at least HIV testing kits and ARVs for mothers and their babies (Health Worker, Mbale Hospital)*.

The need for increased and sustained provision of HIV test kits and antiretroviral drugs for PMTCT was more prominent among health workers at lower level health facilities, as noted.

*Like now we do not have nevirapine. If we get a mother who needs it we can only refer her to Budaka HC IV. If they also do not have it they will refer her to Mbale Hospital and this makes the process very costly for women and their families. Transport by boda boda (motorcycle) to Budaka costs 2 500 Uganda shillings (about 1.4 USD) if the mother has the money she goes. The truth is that most women cannot afford that money. ....these are the things government should address if they want the PMTCT programme to work (Health worker, Health Centre A)*.

Most of the health workers felt frustrated for being criticized by their supervisors, community leaders, pregnant women and their families for the shortfalls in service delivery most of which were beyond their control.

We need government and donors to listen and understand us. We have practical health care needs which frustrate us. Like when we are few health workers, or we do not have nevirapine to give the mothers and their babies what do you do in such cases? The bad thing is that community leaders and every one else expect good services without giving us the needed tools to work (Health worker, Mbale Hospital)

Shortages, especially of HIV testing kits, also constitute missed opportunities to increase male partner HIV testing and involvement in the PMTCT programme.

*We encourage women to come with their partners to test, but there are times when some men come and by bad luck we have no HIV test kits. It frustrates me, because it is really very hard to convince men to come with their wives for antenatal and when we miss them because of shortages it takes us a step behind....( Health worker, Health Centre C)*.

Whereas health workers at TASO were not directly affected by shortages of nevirapine and HIV test kits, they were also concerned that the shortages of supplies at health facilities was a major shortcoming in the PMTCT programme, as a health worker observed.

*At TASO, we counsel and encourage pregnant women in our programme to give birth at the nearby health facilities. But many women, even those who go to health centres for delivery, call us or come here for ARVs for their babies when those drugs are not in hospital. The lack of PMTCT drugs puts an extra burden on these mothers (Health worker, TASO)*.

Interviews with key informants also confirmed the need to strengthen the supply of HIV test kits and ARVs for the PMTCT programme, as one district official observed.

*As a district we have extended PMTCT services, especially HIV testing, to all health centre IIIs. What we need to work on now is to ensure that the supplies for PMTCT are sustained at those health facilities. Shortage of testing kits and nevirapine are common complaints whenever we have PMTCT review meetings. We need a special fund designated for HIV activities rather than relying on donors all the time. Donor projects usually leave gaps when they end which the district cannot fill (District official)*.

Health workers expressed a need to provide *maama kits*, which is a package of basic necessities for use during delivery. These include gloves, polythene sheets, cotton wool, baby sheets, razor blade, threads for tying the umbilical cord etc. The Ministry of Health decided to supply *maama *kits free of charge through the National Medical Stores. At the time of the study, these kits were out of stock and pregnant women at study health facilities were being told to buy maama kits which costs about 8 000-10 000 Uganda shillings (about 4-5 USD) on the open market. Most health workers believed this was a barrier for HIV positive women to deliver at health facilities and access ARVs for their babies since many cannot afford this price.

*We do not have enough gloves, cotton wool and other supplies, so we tell mothers to buy them. We do not like it because we know some of the women cannot afford, but we have to do it .... These supplies should be part of the PMTCT programme (Health worker Mbale Hospital)*.

I think the things we tell mothers to buy discourage them from delivering in hospital yet for the PMTCT programme we need every HIV positive mother to have supervised delivery to reduce chances of HIV transmission. It would be a good addition to the PMTCT package for Government and donors to supply at least some elements of the maama kit like gloves, polythene sheets and cotton wool ..... (Health Worker, Health Centre D)

Health workers from TASO concurred with the need to add maama kits to the PMTCT package as an aspect that would increase the effectiveness of the programme as one health worker noted:

*At TASO we know that most of the HIV positive women are poor and cannot afford to pay for transport to a health facility for delivery, buy drugs and even maama kits. Some mothers in our programme whom we assess and find they cannot afford we give them some of the requirements, like gloves; Government should provide maama kits to all pregnant women including those who are HIV positive (Health worker, TASO Mbale)*.

Indeed, attempts by health workers to build partnerships with their clients to provide the missing supplies were evident during health education talks and the day-to-day running of antenatal clinics as indicated in the explanation by Maria (not real name) a midwife at Mbale Regional Referral Hospital during one of the health education talks,

*Thank you for coming........... One of the things you need to know when you are pregnant is to be prepared for the baby. So you should buy Maama kit which has gloves, at least 2 pairs, two pieces of plastic sheets about 2 metres each, razor blade, cotton wool and remember sheets for the baby etc. If you cannot buy every thing at once buy one item at a time so that by the time you come to give birth you have these requirements. Things have changed, how do you expect us (health workers) to assist you to give birth without gloves? Would you be willing to give birth on a bed which another woman has used without covering it? That is why we want you to buy polythene sheets. I know it is hard for you but it is hard for us health workers as well (Health worker, Mbale Hospital)*.

Some health workers narrated their experiences indicating that despite continuous reminders for women to buy maama kits, some still came for delivery without such kits, largely because they could not afford them.

### Ensure availability of skilled and up-to-date health workers

The need for continuous skills development and up-dating of health workers' knowledge on PMTCT emerged as one of the key areas for strengthening the programme. Most health workers interviewed (21/24) had ever attended training on PMTCT and had been involved in PMTCT activities for 2-5 years (15/24) (Table 1).

However, all health workers in this study expressed the need for more training on PMTCT to update their knowledge and skills given the rapid changes on issues of HIV at global and national levels. The common areas identified for further training were: use of ARVs for babies, ARVs for mothers during and after pregnancy, infant feeding, HIV testing for infants, options to help HIV positive couples to deliver HIV negative children and issues related to discordant couples. The use of CD4 results to decide whether to start a mother on ARVs, disclosure of HIV and other developments like new guidelines and discoveries in areas of HIV prevention, care and support were other training needs expressed by health workers. In view of training needs, health workers noted;

Most health workers have been trained on PMTCT but things are changing very fast. We need regular updates otherwise we shall be challenged by mothers and communities. Some of the issues in PMTCT can be addressed in brief seminars at health units but for major changes, like introduction of new drugs for PMTCT, formal training of health workers is required... (Health worker, Mbale Hospital)

A few health workers had never attended any formal training on PMTCT but had learnt about PMTCT through personal reading and sharing with colleagues at work. These expressed strong need for training, especially covering the use of ARVs for PMTCT which many health workers, especially those from health centres, were not sure of, or were confused about, as indicated in the following quote.

The issue of drugs still puzzles me. I am sure, I am not alone. We received Combivir last week to give pregnant mothers but I am not sure when I should give it to the mother. We have not been trained on use of these drugs (Health worker, Health Centre C)

Another major concern was the need for clarity on infant feeding options for HIV positive mothers.

*Issues of infant feeding should also be addressed in health worker training. Some documents indicate that HIV positive women should continue breast feeding for 6 months while others indicate that breastfeeding for a long time increases the risk of HIV transmission for the baby. What should we tell the HIV positive mothers?. We need more training and reference materials (Health worker, Health Centre C)*.

Findings from key informants also confirmed the need for continuous training of health workers on issues of PMTCT as one district official observed.

*There is more emphasis these days on couple counselling, family planning for HIV positive mothers, use of ARVs during pregnancy and breastfeeding, and the training sessions most health workers attended did not cover these topics. Health workers, often in support supervision visits and PMTCT meetings, have raised this need. We need to conduct district wide refresher training for health workers. The problem we have is that PMTCT guidelines keep changing and we need to re-train health workers time and again but resources are not always readily available to reach all health workers (District official)*.

### Provide support for mothers beyond HIV testing

There was a common feeling among health workers on the need for more support and active follow-up for mothers in the PMTCT programme, especially at government health facilities. Study participants observed that HIV positive mothers require follow-up with supportive counselling to address fears about HIV positive diagnosis, disclosure of HIV status and guidance on infant feeding. Unfortunately, most of the public health facilities in the study area lacked funds for transport and lunch allowance for staff to conduct these follow-up activities. Some health facilities, like the Mbale Hospital in the past, had psychosocial support groups for HIV positive mothers. These groups, however, stopped with the end of a project that was providing funding. In view of this, health workers explained:

*The actual areas for support among women become vivid after women have tested HIV positive but most of the education and counselling in practice focuses on the period before testing. This needs to change so that we (health workers) are provided an opportunity to meet regularly with these women, guide them on disclosure of HIV status, infant feeding... When we had support groups, mothers would meet and share their concerns and advise each other. This was very helpful. Improving the PMTCT programme should have more programmes reaching out to women than what it is now, where we wait for the women to come to us (Health worker, Mbale Hospital)*.

Interviews with health workers at TASO revealed that the PMTCT programme should have a strong link between health facilities and communities so as to meet the needs of HIV positive women and those of their families.

*If health facilities can have, for example, support groups we can link them to our community volunteers and expert clients who can support and encourage women who have just tested HIV positive through use of testimonies (Key informant, TASO Mbale)*.

District officials also observed that whereas the community support interventions for people living with HIV are required, very few donors are willing to fund them.

The challenge we get these days, very few programmes are willing to support community interventions. Every new programe that comes wants to finance health facility based activities yet we need to reach the people where they live and that is where most of the things that hinder PMTCT are located. (District Official)

Another area for support that was emphasized by study participants relates to reaching communities for male partners to take an active role in the PMTCT programme. In view of this, a district official noted:

*One of the major challenges that we need to address in strengthening the PMTCT programme is to reach men, educate them, encourage them to test and be part of the programme. This may not be easy but we need to reach them...Men hold the key to success or failure of the programme (District Official)*.

Health workers emphasized that the PMTCT programme should facilitate them to conduct face to face meetings with communities to educate men and women on the need for men to attend antenatal care with their wives. Interviews with TASO staff revealed that, indeed, men should be, and can be, part of the PMTCT programme.

*TASO has tried to use HIV positive men to reach out to men. Each of the 11 TASO centres has a Positive mens' union (POMU). These are HIV positive men who have come to terms with their diagnosis; they educate communities through radio programmes and music, dance and drama, with messages targeting men (Key informant, TASO)*.

District officials linked some of the gaps with short term projects that support PMTCT programmes.

The challenge we have as a district in the PMTCT programme is the short nature of projects of our partners. Central and local governments need to come up with long term plans and budgets for HIV programmes so that the partners can contribute towards those plans (District Official)

*Government is not doing enough. There should be a conditional grant by Government for HIV. Donor driven programmes leave out some programmes, for example PREFA left out psycho-social support programmes yet they were very helpful (District official)*.

### Ensure adequate integration and universal rollout of PMTCT services

The major sub-themes that emerged under this were: the need to support lower level health centres to provide maternity services and provide ARVs for women in the PMTCT programme. A common concern mentioned by health workers was the need to support the health centre IIIs around Mbale Hospital, to provide maternity services. Health workers revealed that PMTCT services at most health units were limited to HIV counselling, testing, antenatal care and referring women to Mbale Hospital for delivery. As a result, some women preferred to go directly to Mbale Hospital for antenatal care which increased the work load for health workers. At the time of the study, the hospital management had decided that women should attend antenatal care from health centres near them and only go to Mbale Hospital for delivery. Women who came to Mbale Hospital for ANC were being referred back to nearby health centres. The lack of maternity services at health centres was a source of frustration for both mothers and the attending health workers.

*Our mothers get tossed around. They go to Mbale Hospital and they are sent back this way. Yet here we do not conduct deliveries. Some mothers get lost along the way. We should be able to provide all services. (Health Worker, Health Centre B)*.

### Dealing with the challenge of continuing HIV stigma

Another area for improvement mentioned by health workers was the need to address the challenge of continuing HIV stigma. Health workers at lower health centres mentioned that they referred HIV positive women for ARVs to TASO, Mbale Hospital and the Joint Clinical Research Centre (JCRC). Referral of mothers to other HIV care centres made follow-up of mothers and their babies difficult. In addition, most women, especially those who had newly tested HIV positive, were reluctant to go to HIV care centres for fear of stigma.

*We need to provide all the care for preventing HIV transmission to babies and treatment of mothers under the same roof. So that those who fear to go to TASO they are treated by the same health facility, where they are tested and counselled (District Official)*.

*When we refer HIV positive women to TASO for ARVs most do not like it. One woman told us she will not go to TASO, even if it means her dying, she will die. Her main fear was that once she goes to TASO the news about her HIV status will reach her husband whom she feared will mistreat her. (Health worker Health Centre B)*.

### Address heavy work load of health workers

The need for adequate number and right mix of health workers as well as adequate working space emerged as other areas for strengthening the PMTCT programme.

*It is good you have been here with us; you have seen the numbers of women we see and the number of health workers. On average we see 40 new mothers who need HIV counselling and testing and about 20-30 antenatal re-attendees per day. Even the space and time are not enough for us to attend to each mother and give them the best. (Health worker, Mbale Hospital)*.

*...The same health worker is expected to counsel, test, examine the mothers, and fill many registers. It is too much, even a very good health worker cannot offer his/her best in this environment (Health Worker, Health Centre B)*.

Heavy work load was identified as a barrier to utilization of PMTCT guidelines by health workers, as they explained:

*We are always too busy. You cannot get time to read the guidelines. By the time the day ends you are very exhausted (Health worker, Mbale Hospital)*.

*We need simplified and summarized messages like posters for quick reference. (Health worker Health Centre D)*.

Discussions with key informants also revealed that indeed the work load of health workers involved in the PMTCT programme at some health facilities was high and more health workers were required for quality provision of health services.

## Discussion

We explored the experiences and lessons learnt by health workers on how to improve the delivery of PMTCT services in Mbale District, eastern Uganda. The findings revealed the need to ensure constant supply of critical PMTCT supplies including HIV test kits and ARVs for prophylaxis for both mothers and their babies as a key requirement to strengthen the programme. An earlier study based on the pilot phase of the PMTCT programme conducted in 2003 in Uganda also highlighted the need to address constraints related to shortage of supplies for the successful implementation of the programme[10]. In consonance with our findings, a retrospective analysis study done at Mbale Hospital, based on antenatal data for a seven year period also highlighted the challenge of stock outs of HIV test kits in utilization of antenatal based HIV counselling and testing [22] as an entry point in the PMTCT programme. Similarly, Medley and Kennedy, in a study carried out in Central Uganda, also documented frequent stock-out of supplies as a major barrier to the implementation of antenatal provider-initiated HIV testing [23]. Stock-out of HIV test kits and ARVs for the PMTCT programme mirrors the general health system inadequacies within which the PMTCT programme is being implemented and have been reported in other African countries [24]. It is thus not surprising that most of such interventions are sub-optimal in performance [25]. Shortage of PMTCT supplies was a source of frustration for both health workers and HIV positive women. To women, these shortages often translated into additional costs in the form of transport as they struggle to find ARVs for themselves and for their babies. The government and partner agencies should prioritise the critical supplies for PMTCT for an effective programme. The persistent shortages of PMTCT supplies also indicate barriers in translation of research findings into action plans to address such inadequacies.

Our findings further revealed the need for government and other actors in the PMTCT programme to consider provision of *Maama *kits (hospital requirements for use during delivery) as part of the PMTCT programme so as to increase health facility delivery. This addition is justifiable given a multiplicity of needs that HIV positive women have in the context of poverty. The Uganda National Household survey 2009/10 revealed that 25% of the Ugandans were poor-living in households below the poverty line (less than one USD dollar a day). In the eastern region, where Mbale is located, poverty was estimated at 24%[26]. Although attempts are made by the Ugandan Ministry of Health to supply *maama *kits to health centres, the supplies are often very inadequate and irregular. These findings concur with those of a study done in two districts of eastern Uganda where stock out of *maama *kits was a common occurrence and a barrier for women to deliver at health facilities [27]. Provision of maama kits has a dual benefit 1) as an equity promotion measure to enable the poor to deliver at health facilities and 2) as an entry point to integrate and promote linkages between PMTCT, maternal and child health, and sexual and reproductive health. Indeed, the World Health Organization PMTCT strategic vision 2010-2015 stresses the need for this linkage [1]. In addition, universal provision of maama kits is likely to have great benefit in reducing child mortality and improving maternal health (MDG 4 and 5) where Uganda is not doing well [28,29].

Whereas the PMTCT programme has been extended to lower level health facilities [4], our findings show an urgent need for more efforts in keeping health workers abreast with the rapid changes in knowledge in the field of HIV owing to new evidence and the subsequent changes in national and global policy recommendations. At some health centre IIIs, use of ARVs for PMTCT prophylaxis had been introduced, however, most health workers, especially those at lower level health facilities expressed need for training on use of these drugs. This may translate into a national wide need, as the country revises the national PMTCT guidelines in view of the 2009, World Health Organization advice on use of antiretroviral drugs for PMTCT and infant feeding [30]. The Uganda National PMTCT guidelines were issued in 2003 after the pilot phase of the PMTCT programme [31] and have been revised several times, most recently in 2010 [32] following the WHO guidance [30]. Our findings depict the continuous struggles of frontline health workers to provide quality PMTCT services in the context of changing global and national policy guidelines, yet without matched efforts to ensure that health workers are fully orientated on the new guidelines. The fact that some health facilities in our study had new PMTCT drugs like Combivir, yet health workers at some of those health facilities had not been oriented on use of such drugs is an example of how challenging it is to translate policies into practice in resource limited settings. It is also a pointer on the need by programme stakeholders, including international donor agencies, to appreciate the intensity of adjustments needed at health facilities whenever policy guidelines change. Whereas full training packages may be required in case of major changes in the programme, low cost training events in the form of small groups at health units, may help to bridge some of the existing knowledge gaps among health workers. Urgent knowledge gaps identified were: infant feeding counselling, use of ARVs during pregnancy and breastfeeding by mothers and their infants, and discordance among other gaps. The knowledge gaps among health workers on use of ARVs and infant feeding options in our study are not surprising given that these components of the PMTCT programme have been a constant subject of change.

Study findings revealed a need to provide more support for HIV positive women beyond HIV testing, to one which would include post-test counselling and guidance which could take the form of a psycho-social support group or individual support through home visits. Whereas the Uganda national counselling and testing guidelines recommend post test clubs as a source of support for HIV positive mothers, this support was generally lacking at most health facilities. Most of the key informants indicated that such groups were available at the start of the PMTCT programme and were beneficial to mothers in dealing with different challenges. It was a common occurrence that such groups had stopped when project funding ceased. There is need for programme appraisal to ensure that the necessary elements, especially those that support women beyond the clinic, are not lost as the programme is being extended to lower level health facilities and when project support comes to an end. The role of support groups for women living with HIV in high prevalent settings, such as Uganda, has also been highlighted by Medley and colleagues as a necessary strategy enabling women to develop skills to cope with a life changing diagnosis[33] more so for those who discover, during pregnancy, that they are HIV positive.

A particular concern among health workers was the need for more integrated service delivery as a building block for the PMTCT programme, especially at lower level health facilities. Facilitating health centres around Mbale hospital to provide maternity services was identified as one way that would increase the effectiveness of the PMTCT programme. This could help to reduce congestion at the hospital and provide an opportunity for women to build relationships and trust with health workers who will assist them at the time of delivery and in responding to individual concerns of HIV positive women. Providing ARVs and septrin to HIV positive women in the antenatal care clinic is needed to protect women from stigma as they come to terms with the HIV diagnosis. Indeed, the 2010 WHO PMTCT strategic plan highlights the need to integrate PMTCT services with maternal, newborn and child health and sexual and reproductive health programmes [1].

Marked differences were noted between the TASO PMTCT programme and the regular government PMTCT programme at public health facilities. Whereas the TASO programme is less affected by shortages of PMTCT supplies, the newly tested HIV positive women tended to avoid TASO for fear of stigma. Thus most of the newly tested HIV positive women, preferred to obtain HIV care from the health facilities which offer other general services, yet most of the public health facilities are faced with regular stock-out of such drugs and indeed some health facilities were not providing such care. This finding shows a real example where persistent HIV stigma together with limited integration of HIV services remain key challenges to timely access of HIV care in Uganda.

Other areas for improvement that emerged in this study were: provision of adequate numbers and cadres of health workers, adequate space for counselling and provision of simplified reference materials on PMTCT. The need to reach men through community programmes and the media was also critical. The role of a conducive environment in having an effective PMTCT programme was documented at the start of this initiative [10]. Mixed methods studies conducted in Mbale District in 2003 and 2005 also highlighted inadequate resources, minimal staff, inadequate training and follow-up of clients as barriers to infant feeding counselling [34] in the context of PMTCT. Another study conducted at 10 clinics in central Uganda also highlighted lack of adequate space for counselling, frequent stock-out of supplies and shortage of counsellors as some of the health system barriers affecting implementation of provider-initiated HIV counselling and testing in Uganda [23]. Similar challenges have been documented in Tanzania [35,36]. These findings point at the urgent need to strengthen the health systems as part of the social context within which the PMTCT programme operates for better results. Health workers in our study revealed that often the inappropriate environment in which they work, characterized by shortage of critical supplies, strained their relationship with women. The shortages in hospital supplies, with which women and their families are faced, represent a health policy contradiction in Uganda, where health services are presumed to be free at public health facilities. Health workers in our study felt they needed to be listened to and to be understood, particularly with regard to the day-to- day constraints they encounter in the delivery of maternal and child health services including PMTCT. Shortage of supplies persisted despite continued submission of reports and requests by health facilities indicating the need for a more proactive approach to addressing health system challenges. Indeed these challenges pose a threat to the likely implementation and success of the revised WHO PMTCT guidelines [30].

Findings of our study should be interpreted in light of the following limitations; 1) given the qualitative nature of our study we are unable to provide quantifiable indicators on health system gaps affecting the PMTCT programme. 2) Since we interviewed health workers involved in the programme, some responses may have been biased. However, triangulation of data from different sources including observations during the PMTCT clinic days helped to improve the trustworthiness of study findings. We present largely health worker perspectives. Future studies should address perspectives of service users to better understand what more needs to be done to strengthen the PMTCT programme. We conducted the study largely in public health facilities; transferability of our findings to other public health facilities in Uganda is possible. However, our findings may not apply in the not for profit or private health facilities whose constraints and priorities may be different.

## Conclusions

The voices of health workers in this study revealed that health workers are and should be treated as key stakeholders in the design, implementation and strengthening of the PMTCT programme in Uganda. In addition these findings reflect the need to address the practical challenges health care providers are confronted with in the delivery of PMTCT services. It is imperative that government, civil society organizations and donors for the PMTCT programme address the challenges of shortage of PMTCT supplies (HIV test kits and ARVs), continuous health worker training, heavy work load, follow-up and support for mothers, as urgent needs to strengthen the PMTCT programme. Real hope in curtailing the spread of HIV through mother-to- child transmission in Uganda lies in strengthening health systems to better deliver the prevention package for PMTCT. Our study challenges the national and global actors in HIV prevention, to ensure that policy planning and intervention match realities on the ground and do not become thwarted by stock outs, inadequately trained health workers and the like as the health workers in this study have revealed.

## Abbreviations

AIDS: Acquired Immune Deficiency Syndrome; ANC: antenatal clinic; ART: Antiretroviral Therapy; ARVs: antiretroviral drugs; CDC: Centers for Disease Control and prevention; CIFF: Children's Investment Fund Foundation; HC: Health Centre; HIV: Human Immune-deficiency Virus; IDC: infectious diseases clinic; JCRC: Joint Clinic Research Centre; MDG: Millennium Development Goals; MTCT: mother-to-child transmission; NGOs: Non Government Organisation; NVP: Nevirapine; PEPFAR: Presidential Emergence Plan for AIDS Relief; PMTCT: Prevention of mother-to-child transmission of HIV; POMU: Positive Mens' Union; PREFA: Protecting Families Against HIV/AIDS; RCT: Routine HIV counselling and testing; TASO: The AIDS Support Organisation; UAC: Uganda AIDS Commission; and VCT: voluntary HIV counselling and testing.

## Competing interests

The authors declare that they have no competing interests.

## Authors' contributions

JR conceived the study. All authors (JR, JKT, TT, SN & HKH) participated in the design of the study. JR participated in data collection, JR and HKH participated in data analysis. All authors (JR, JKT, TT, SN & HKH) participated in the interpretation, writing of the manuscript and approved it for submission.

## Pre-publication history

The pre-publication history for this paper can be accessed here:

http://www.biomedcentral.com/1472-6963/12/3/prepub
